# The critical role of complete blood count in the management of patients with COVID-19

**DOI:** 10.11604/pamj.supp.2020.35.2.23764

**Published:** 2020-06-04

**Authors:** Maryame Ahnach, Nouama Bouanani, Sara Nejjari, Mounia Bendari, Kamal Doghmi, Chafik El kettani

**Affiliations:** 1 Department of Hematology, Cheikh khalifa International University Hospital, Mohammed VI University of Health Sciences Casablanca, Morocco; 2 Department of Hematology, Military Hospital Mohammed V, Mohammed V University Faculty of medicine and Pharmacy, Rabat, Morocco; 3 Department of Anesthesiology and Reanimation, Cheikh khalifa International University Hospital, Hassan II VI University Faculty of Medicine and Pharmacy, Casablanca, Morocco

**Keywords:** COVID-19, complete blood count, hematology abnormaly

## To the editors of the Pan African Medical Journal

Since March 2020, the world has declared a global health crisis caused by the coronavirus COVID-19 pandemic. After Asia, Europe, the United States is the most affected. The main features described are pulmonary manifestations, however, this systemic infection seems to have a direct impact on the hematopoietic system. Many publications have documented the clinical, biological and radiological characteristics of COVID-19 infection, and several international societies have developed protocols for management and follow-up. In these recommendations, biological analysis and especially the complete blood count, represents a major tool in the diagnosis, monitoring, detection of severe forms, and hematological complications [1]. Quantitative hematologic abnormalities have been reported since the first papers, all blood cells can be affected during COVID-19, mainly leukocyte and platelet cells [2].

During asymptomatic forms or the incubation period, patients can manifest a moderate abnormality in the blood count, Qilin et al suggested the potential value of eosinopenia as predictors of early identification of COVID-19 [3]. Regarding symptomatic forms, Guan et al found in a large series of 1099 cases, a predominance of lymphopenia (83%), thrombocytopenia (36.2) and neutropenia (33.7%) [4]. In severe cases, these abnormalities were more prominent (96.1% versus 80.4% lymphopenia, 57.7% versus 31.6% thrombocytopenia and 61.1% versus 28.1% for leukopenia) [5]. Lymphopenia is the most common sign, in fact, the coronavirus attack directly and indirectly the lymphocytes by immune and inflammatory mechanisms [6]. The analysis of the lymphocyte count is therefore a reliable indicator of the severity, which can be really useful in the monitoring and therapeutic adaptation, moreover after clinical improvement the lymphocyte count is corrected [7].

Among recent studies, a meta-analysis, have reported the association between low platelet count (less than 30 10^9^/l) and the increased risk of severity and mortality from COVID-19. The pathophysiologic mechanism of thrombocytopenia is multifactorial due mainly to disseminated intravascular coagulation, micro-vascular thrombosis and macrophagic activation syndromes, that can cause bleeding and poor outcome [8]. In summary, the blood count as a routine biological analysis, keeps a prominent role in the early diagnosis and follow-up of COVID-19 infection. The blood cells perturbations are seen as a prognosis factors, careful analysis and interpretation of lymphocyte and platelet count, allows not only to evaluate the prognosis, but above a clinician to adapt therapeutic care. Compared to specific inflammatory biomarkers tests (Lactate dehydrogenase, Interleukin, procalcitonin, etc.), the blood count remains a less expensive alternative, especially in countries with limited resources.

## Competing interests

All authors declare no competing interests.

## References

[1] Lippi G, Plebani M (2020). Laboratory abnormalities in patients with COVID-19 infection. Clin Chem Lab Med.

[2] Fan BE, Chong VCL, Chan SSW, Lim GH, Lim KGE, Tan GB (2020). Hematologic parameters in patients with COVID-19 infection. Am J Hematol.

[3] Li Q, Ding X, Xia G, Geng Z, Chen F, Wang L (2020). A simple laboratory parameter facilitates early identification of COVID-19 patients. medRxiv.

[4] Guan WJ, Ni ZY, Hu Y, Liang W, Chun-Quan Ou, Jian-Xing He (2020). Clinical Characteristics of Coronavirus Disease 2019 in China. N Engl J Med.

[5] Terpos E, Ntanasis-Stathopoulos I, Elalamy I, Kastritis E, Sergentanis TN, Politou M (2020). Hematological findings and complications of COVID-19. Am J Hematol.

[6] Tan Li, Wang Qi, Ding DZJ, Huang Q, Tang Y (2020). Lymphopenia predicts disease severity of COVID-19: a descriptive and predictive study. Signal Transduct Target Ther.

[7] Wang F, Nie J, Wang H, Zhao Q, Xiong Y, Deng L (2020). Characteristics of peripheral lymphocytes subset alteration in COVID-19 pneumonia. J Infec Dis.

[8] Lippi G, Plebani M, Henry BM (2020). Thrombocytopenia is associated with severe coronavirus disease 2019 (COVID-19) infections: A meta-analysis. Clin Chim Acta.

